# Preliminary Evaluation of Clinical and Angiographic Outcomes with Biodegradable Polymer Coated Sirolimus-Eluting Stent in* De Novo* Coronary Artery Disease: Results of the MANIPAL-FLEX Study

**DOI:** 10.1155/2016/9324279

**Published:** 2016-08-14

**Authors:** Ranjan Shetty, Jayesh Prajapati, Umesh Pai, Kiran Shetty

**Affiliations:** ^1^Kasturba Medical College and Hospital, Manipal, Karnataka 576104, India; ^2^Apollo Hospitals International Limited, Gandhinagar, Gujarat 382428, India

## Abstract

*Objective*. The objective of the MANIPAL-FLEX study was to evaluate the feasibility, preliminary safety, and efficacy of the Supraflex sirolimus-eluting stent (SES) implantation, in* de novo* coronary artery disease, using clinical and quantitative coronary angiography (QCA) follow-ups.* Methods*. This was a prospective, nonrandomized, multicenter, single-arm study that enrolled 189 patients with* de novo* coronary artery disease who were treated with the Supraflex SES. Of 189 patients enrolled, the first 61 consecutive patients who consented to a 9-month follow-up evaluation by QCA, irrespective of presence of symptoms, were to be followed up with angiography at 9 months. The primary endpoint of the study was target lesion failure (TLF), including cardiac death, myocardial infarction, and target lesion revascularization during 12-month follow-up after the index procedure.* Results*. The mean age of the study population was 58 ± 11 years, with 51.3% (97/189) of hypertensive patients. Total of 66 lesions, analyzed by offline QCA, showed good scaffolding of the target vessel with in-stent late lumen loss at 9 months of 0.18 ± 0.23 mm. The observed TLF at 30-day, 6-month, and 12-month follow-up were 2 (1.1%), 6 (3.2%), and 10 (5.3%), respectively.* Conclusion*. This study provides preliminary evidence for the feasibility, safety, and efficacy of the Supraflex sirolimus-eluting stent.

## 1. Introduction

In spite of the reduction of restenosis and the need for repeat revascularization with drug-eluting stents (DESs), the persistence of adverse events with first-generation durable polymer-based DES enlightens the opportunity for further improvement in DES technology [1–3]. The potential link between the durable polymer and late adverse events has prompted the development of biodegradable sources of drug delivery to a stented vessel [4–6]. The use of a biodegradable polymer has the potential to reduce the sustained inflammatory response of the arterial wall, facilitating reendothelialisation and minimising the risk of thrombus formation and late restenosis [7, 8]. Furthermore, first-generation DESs, with thick-strut design, are associated with more thrombotic events than thin-strut comparators in* ex vivo* and experimental models [9]. To resolve these issues, newer DES technologies, such as the use of stents with thinner struts and the use of biodegradable coatings to improve biocompatibility, have been developed. These properties might confer an additional benefit in terms of reendothelialisation and restenosis [10, 11].

The design and engineering of stent platforms is constantly evolving with major advancements made over the years [12–14]. Furthermore, stent design including strut thickness, metal composition, and radial strength can each influence short- and long-term clinical outcomes [15, 16]. The Supraflex [Sahajanand Medical Technologies Pvt. Ltd. (SMT), India] was developed using the same proven biodegradable polymer-based, sirolimus-eluting technology as on the Supralimus-Core (SMT, India) sirolimus-eluting stent (SES) but was applied to the newer Flexinnium (SMT, India) cobalt-chromium bare-metal stent. This new stent design has thinner struts (60 *μ*m) with highly flexible “S-link.” It has been considered that such stent design could facilitate deliverability, especially in complex lesions, without compromising safety and efficacy. The MANIPAL-FLEX study was designed to evaluate the feasibility, preliminary safety, and efficacy of the Supraflex SES implantation, in* de novo* coronary artery disease (CAD), using clinical and quantitative coronary angiography (QCA) follow-ups.

## 2. Materials and Methods

### 2.1. Study Design and Patient Population

**Figure 1:**
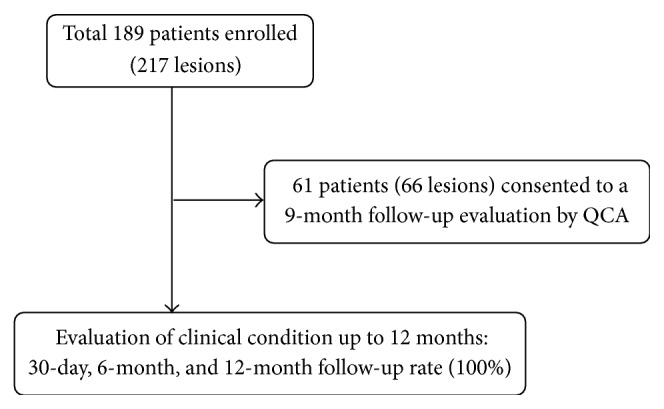
Flow chart of the study.

This was a prospective, nonrandomized, multicenter, single-arm study which enrolled 189 patients with* de novo* CAD, who were treated with the Supraflex SES, between November 2012 and March 2014. Of 189 patients enrolled, the first 61 consecutive patients who consented to a 9-month follow-up evaluation by QCA, irrespective of presence of symptoms, were to be followed up with angiography at 9 months. Clinical follow-up was intended at 30 days, 6 months, and 12 months for all enrolled patients (Figure 1). Clinical inclusion criteria were age ≥18 years, symptoms of angina or ischemia, and acceptable candidacy for coronary artery bypass surgery. Angiographic criteria included* de novo* coronary lesions ≤44 mm in length, reference diameter of 2.0 to 4.5 mm, stenosis ≥50%, and agreement to undergo all protocol-required follow-up examinations including angiographic follow-up at 9 months. Exclusion criteria were cardiogenic shock, left ventricular ejection fraction <30%, ostial lesion location, major surgery within 6 weeks before planned stenting procedure, stenosis >50% of the left main coronary artery, angiographic evidence of thrombus or poor distal flow at the lesion site, lesion at a junction of a side branch with a diameter >2 mm, and staged procedure planned within 3 months of the index procedure. The study was reviewed and approved by institutional ethics committees [Kasturba Hospital, Manipal (IEC 471/2012), and Apollo Hospitals International Ltd. (ECR/30/Inst/GJ/2013)]. The study was conducted in accordance with the principles of good clinical practice and the Declaration of Helsinki. Patients' participation in the study was voluntary and eligible patients signed written informed consent prior to the interventional procedure.

### 2.2. Study Device

**Figure 2:**
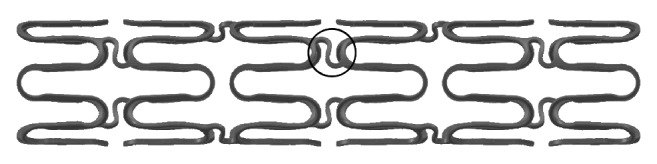
Supraflex sirolimus-eluting stent: a schematic view of the stent structure (circle shows flexible “S-link”).

**Figure 3:**
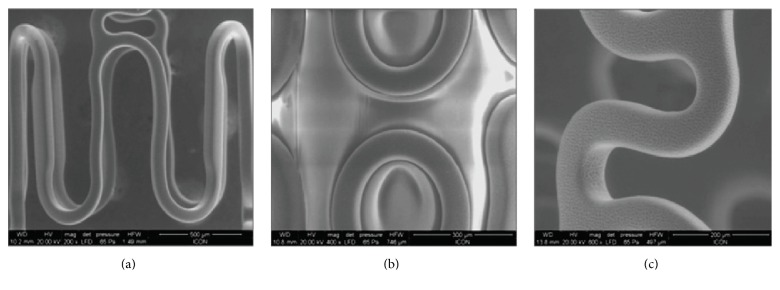
Scanning electron microscopy (SEM) images of a (a) normal, (b) crimped, and (c) expanded Supraflex sirolimus-eluting stent.

**Figure 4:**
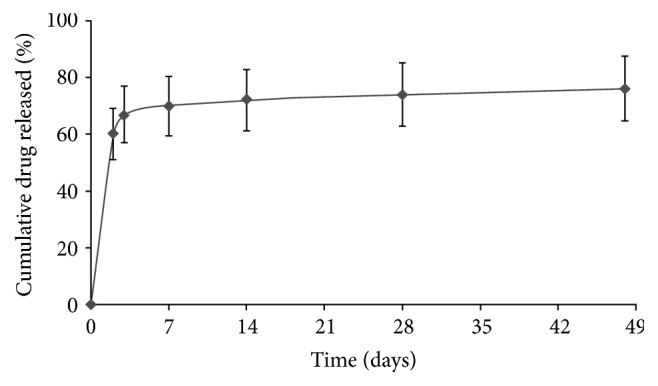
Drug release kinetics of the Supraflex sirolimus-eluting stent.

The Supraflex SES is designed, using the Flexinnium bare-metal stent as a platform, with highly flexible “S-link” (Figure 2). The Supraflex SES has L605 cobalt-chromium (Co-Cr) alloy, having strut thickness of 60 *μ*m with biodegradable polymers and drug load of 1.4 *μ*g/mm^2^. The stent is mounted on a rapid-exchange catheter with a high-pressure, semicompliant balloon. The coating layer comprises drug sirolimus blended together with biodegradable polymeric matrix. This matrix includes different biodegradable polymers, Poly(L-lactide), 50/50 Poly(DL-lactide-co-glycolide), and Polyvinylpyrrolidone, to control the drug elution from stent coating. The average coating thickness of the Supraflex SES is between 4 and 5 *μ*m. The coating of the polymer is conformal around the stent struts and not limited to abluminal surface of the stent.  Figure 3 shows scanning electron microscopy (SEM) images of a normal, crimped, and expanded Supraflex stent. These images demonstrate that the surface of the stent was coated uniformly with a thin film that conformed faithfully to the stent surface, the contours of the stent struts, and the balloon assembly. About 70% of drug is released within 7 days and remaining drug is released over a period of 48 days (data on file at SMT, India) (Figure 4). The polymers retain their properties for a limited period and then gradually degrade into biologically acceptable molecules that are metabolized and removed from the body via normal metabolic pathways. This process takes about 9–12 months. The Supraflex SES is available in diameters of 2.0, 2.25, 2.5, 2.75, 3.0, 3.50, 4.0, and 4.5 mm and in length sizes of 8, 12, 16, 20, 24, 28, 32, 36, 40, 44, and 48 mm.

### 2.3. Coronary Intervention Procedures and Adjuvant Medications

Coronary interventional procedures and adjuvant medications were performed according to standard guidelines [17]. All patients received dual antiplatelet therapy (DAPT) including a loading dose of aspirin (300 mg) and clopidogrel (600 mg) or prasugrel (60 mg) or ticagrelor (90 mg). The procedural anticoagulation was achieved either with heparin or with bivalirudin. However, the intraprocedural administration of glycoprotein IIb/IIIa inhibitor was at the investigator's discretion. All patients were advised to maintain DAPT (aspirin; 75–300 mg daily indefinitely and clopidogrel; 75 mg daily or prasugrel; 10 mg daily or ticagrelor; 90 mg twice daily for at least 12 months) after the procedure.

### 2.4. Patient Follow-Up

Clinical data were collected before and after the index procedure and recorded in a dedicated database for all patients. Total 61 patients underwent angiographic evaluation at 9 months. Clinical/telephonic follow-up was scheduled for all enrolled patients at 30 days, 6 months, and 12 months, and collected details like assessment of angina status, monitoring of cardiovascular and antithrombotic drug use, interim hospitalisations, occurrence of major adverse cardiac events, and any invasive and noninvasive diagnostic test or interventional treatment that had occurred since the previous contact. An independent clinical events committee (CEC) reviewed and adjudicated all major adverse cardiac events.

### 2.5. Endpoints of the Study and Definitions

The primary endpoint of the study was target lesion failure (TLF), including cardiac death, myocardial infarction (MI), and target lesion revascularization (TLR) during 12-month follow-up after the index procedure. Secondary endpoints included acute success (lesion success, device success, and procedural success) and in-stent late lumen loss at 9 months by QCA. Lesion success was defined as a final diameter stenosis <30% with any device with TIMI 3 flow; device success was defined as stenosis of <30% diameter with TIMI 3 flow in the absence of device malfunctions; and procedure success was defined as a final diameter stenosis <30% with TIMI 3 flow, without the occurrence of death, MI, or repeat revascularization of the target lesion during the hospital stay.

Any death was considered cardiac, unless clear noncardiac causes could be determined. MI was defined by elevation of cardiac troponin (cTn) values [>5 × 99th percentile upper reference limit (URL)] in patients with normal baseline values (≤99th percentile URL) or a rise of cTn values >20% if the baseline values are elevated and are stable or falling. TLR was defined as repeat percutaneous intervention of the stented lesion including 5 mm proximal and distal from the edge of the stent or bypass surgery of the target vessel that was performed for a clinical indication and was due to restenosis or closure of the target lesion. Target vessel revascularization (TVR) was defined as repeat revascularization by either percutaneous coronary intervention (PCI) or CABG of the target vessel. We also evaluated the incidence of stent thrombosis (ST) as a safety endpoint during 12-month follow-up period, as defined by the Academic Research Consortium (ARC) [18].

### 2.6. Angiographic Assessments

Angiographic assessments were performed at baseline, after procedure, and at 9-month follow-up, in two orthogonal views after the intracoronary administration of nitroglycerin. A quantitative offline coronary angiographic analysis was performed [Coronary Angiography Analysis System (CAAS, version 5.9.2); Pie Medical Imaging, Maastricht, Netherlands] with automatic edge detection by senior interventional cardiologist who had experience in reading coronary angiograms with performing QCA and was blinded to the clinical data. For every patient, in-stent (bordered by the stent margins) and in-segment (in-stent region plus 5 mm margins proximal and distal to the stent) measurements were performed by QCA. The quantitative angiographic parameters including reference vessel diameter (RVD), percentage diameter stenosis (DS), minimal lumen diameter (MLD), late luminal loss, binary restenosis, and acute gain were evaluated. Percent diameter stenosis was defined as 1 − [MLD/RVD] × 100, late luminal loss was defined as the difference between the MLD at the completion of the stenting procedure and the MLD measured at angiographic follow-up, binary restenosis was defined as a diameter stenosis of ≥50% at angiographic follow-up, and acute gain was defined as the MLD immediately after the procedure minus the MLD before the procedure.

### 2.7. Statistical Analysis

The MANIPAL-FLEX study was designed to provide adequate preliminary data about the safety and efficacy of the Supraflex SES and was therefore considered explorative. Resulting values will be the basis for sample size calculations for future studies. Based on similar studies with other drug-eluting stent systems, a sample size of 189 patients (61 patients for angiographic assessments) was expected to be sufficient for preliminary evaluation of the safety and efficacy of the device. Data are presented using descriptive statistical methods. Continuous variables were presented as mean ± standard deviation, whereas categorical variables were expressed as percentages. All data were processed using the Statistical Package for Social Sciences, version 15 (SPSS, Chicago, IL, USA).

## 3. Results

### 3.1. Baseline, Lesion, and Procedural Characteristics

The baseline clinical characteristics are outlined in Table 1. Among 189 enrolled patients, the mean age was 58 ± 11 years and 30.7% (58/189) had diabetes mellitus. Lesion and procedural characteristics are listed in Table 2. For patients undergoing examination by quantitative coronary angiography (*n* = 61) at 9 months, the mean lesion length was 9.70 ± 6.94 mm, and the average RVD was 2.42 ± 0.49 mm (Table 3). Among overall population most lesions (48.4%; 105/217) were located in the left anterior descending artery and 181 (83.4%) of 217 lesions were type B2/C, according to the American College of Cardiology/American Heart Association classification scheme. Lesion, device, and procedural success were achieved in 100% of patients.

### 3.2. Quantitative Coronary Angiography Outcomes

**Figure 5:**
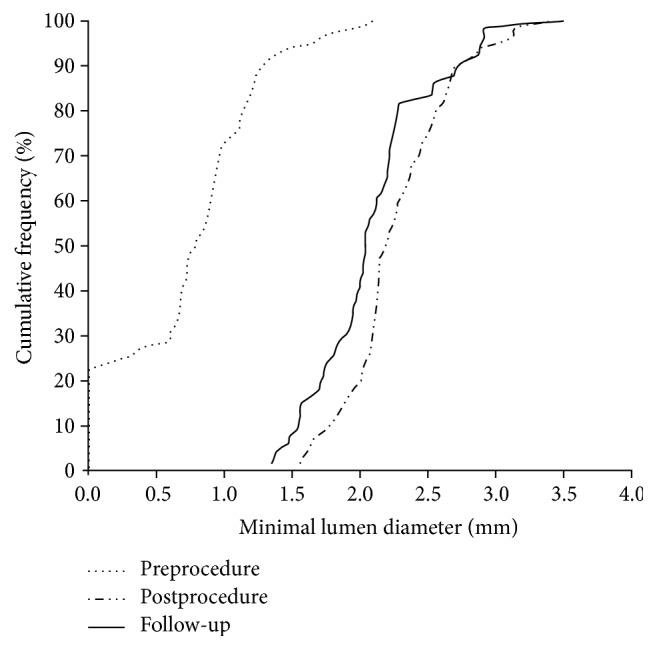
Cumulative frequency distribution curves of in-stent minimal lumen diameter by QCA.

Pre- and postprocedural and 9-month QCA measurements were performed on the first 61 consecutive patients (66 lesions) who consented to a 9-month follow-up evaluation by QCA, irrespective of presence of symptoms, and are presented in Table 3. The mean in-stent late lumen loss at 9 months was 0.18 ± 0.23 mm, while the mean in-segment late lumen loss was 0.11 ± 0.33 mm. In-stent diameter stenosis after 9 months was 16.14 ± 7.52 percentage with binary restenosis rate of 1.5% (1/66).  Figure 5 demonstrates the cumulative frequency distribution curves of in-stent MLD at preprocedure, postprocedure, and 9-month follow-up.

### 3.3. Clinical Outcomes

The in-hospital incidence of cardiac death, MI, TLR, TVR-nontarget lesion, and/or stent thrombosis was 0%. Clinical follow-up up to 12 months was completed in all 189 patients (100%) and is summarized in Table 4. No TLR was observed during the first 30 days, though from 30 days to 6 months, 2 patients (1.1%) had TLR and from 6 months to 12 months, another two patients had TLR, resulting in a 12-month cumulative TLR of 2.1% (4/189). The observed TLF at 30-day, 6-month, and 12-month follow-up were 2 (1.1%), 6 (3.2%), and 10 (5.3%), respectively. According to ARC, there was only one case (0.5%) of late stent thrombosis and was recorded 11 months following the index procedure. One patient died from a new MI during 30-day follow-up and another two sudden deaths were noted at 6-month and 12-month clinical follow-up with cause of death adjudicated as cardiac death but without MI or stent thrombosis. A total of 98.9% and 73.7% of patients were on DAPT at 6 months and 12 months, respectively.

## 4. Discussion

Due to concern related to impaired healing and inflammation associated with first-generation durable polymer DES, scientific interest has been created in developing newer devices, with improved polymer and metallic platform technologies [1, 19, 20]. Thus, the concept of thin-strut, biodegradable polymer coated DES emerged in the recent years. Biodegradation ensures that both the drug and coating are absorbed from the stent surface after their function is accomplished, leaving only a metal stent covered by neointima and endothelium without further continuous irritation of the arterial wall [21]. Moreover, the randomized LEADERS studies showed that biodegradable polymer stents reduced the risk of cardiac events associated with very late stent thrombosis, which might improve long-term clinical outcomes compared with durable polymer sirolimus-eluting stents [22, 23].

Furthermore, in clinical circumstances, thin-strut stents have been shown to produce less neointimal hyperplasia than thick-strut bare-metal stents [10, 11]. Also, several clinical studies using thin-strut bare-metal cobalt-chromium stents have shown the advantages of enhanced visibility, deliverability and radial strength, and reducing restenosis when compared with bare-metal stainless steel stents [24–27]. In this context, the Supraflex SES was developed using the same proven biodegradable polymer-based, sirolimus-eluting technology as on the Supralimus-Core SES but was applied to the thinner struts (60 *μ*m) cobalt-chromium stent platform (Flexinnium bare-metal stent). The clinical safety and effectiveness of the Supralimus-Core was already established in various clinical studies [28–34].

In this MANIPAL-FLEX study, patient population had higher rates of hypertension (51.3%), hypercholesterolemia (32.3%), type B2/C (83.4%) lesions, and total occluded (31.8%) lesions compared to real-world Supralimus-Cores' S-CORE registry and CORE registry [32, 34]. The combination of these factors makes the patient population for this study unusually complex. Regardless of this individuality, the results of this experience confirm the excellent acute performance of the Supraflex SES. The ease of deliverability, flexibility, and crossing of the Supraflex SES across the lesion was confirmed with 100% lesion, device, and procedural success, which preliminarily indicates feasibility for this device.

Additionally, in-stent late lumen loss at 9-month poststent implantation was one of the secondary endpoints of the MANIPAL-FLEX study. The main aim to select this endpoint was that late lumen loss as an angiographic factor is confirmed to be a dependable predictor of the long-term clinical efficacy of DES and also strong predictor of binary restenosis and TLR [35]. The 9-month in-stent late lumen loss of 0.18 ± 0.23 mm observed in the current study situates Supraflex SES among the most effective DES systems, which resembles to the late lumen loss reported for other DESs using limus drugs such as Promus Element (0.15 mm), Xience (0.12 mm), Biomatrix (0.26 mm), Nobori (0.10 mm), or Synergy (0.10 mm) [36–39]. The observed late lumen loss, in this study, with the Supraflex SES translated into an unpretentious level of binary in-stent restenosis (1.5%) and binary in-segment restenosis (1.5%) which is also comparable to sirolimus-eluting Ultimaster stent [40].

In this study, the primary outcome, 5.3% TLF rate at 12 months, compares well to the 5.1% TLF rate reported for the Orsiro DES in the BIOFLOW-III registry [41]. Furthermore, it is noted that, at 12 months, the TLR rate in the MANIPAL-FLEX study was 2.1% which is lower to the rate of TLR with the Ultimaster DES (3.8%) at one year [40]. Also, analysis of 50 patients from the MANIPAL-FLEX study, presented at the Euro-PCR meeting 2015, showed good clinical outcomes [42]. The promising clinical outcomes in this study might be attributed to the biodegradable polymer and the unique design of the Supraflex stent. In summary, this study provided preliminary evidence for the feasibility, safety, and efficacy of the Supraflex sirolimus-eluting stent in the treatment of* de novo* CAD.

### 4.1. Limitations of the Study

This study is limited by the fact that it is a single-arm, nonrandomized study without comparator or adequate statistical power for conclusive definition of angiographic outcomes. In addition, limited clinical outcomes up to 12 months may not be enough to capture all the adverse events, especially very late stent thrombosis. Accordingly, these results must be measured in a comparative study with larger population, over longer follow-up duration.

## 5. Conclusion

This MANIPAL-FLEX study provided preliminary evidence for the feasibility, safety, and efficacy of the Supraflex SES. In this study, the Supraflex, thin-strut biodegradable polymer coated sirolimus-eluting stent demonstrated high-level efficacy, by the relatively low late lumen loss, at 9-month angiographic follow-up. Also, implantation of the Supraflex SES was proven to be safe, with high acute lesion, procedure, and device success rates. A randomized study with larger study population and over long-term follow-up is needed for further information and validation.

## Figures and Tables

**Table 1:** Baseline clinical characteristics for the entire population (*n* = 189) and for patients undergoing examination by quantitative coronary angiography (*n* = 61) at 9 months.

Variables	*n* = 189	*n* = 61
Age, (mean ± SD, years)	58 ± 11	56 ± 10
Male, *n* (%)	136 (72.0%)	49 (80.3%)

*Cardiovascular risk*		
Diabetes mellitus, *n* (%)	58 (30.7%)	13 (21.3%)
Hypertensive, *n* (%)	97 (51.3%)	26 (42.6%)
Hypercholesterolemia, *n* (%)	61 (32.3%)	18 (29.5%)
Current smoker, *n* (%)	45 (23.8%)	11 (18.0%)
Family history of CAD, *n* (%)	12 (6.3%)	4 (6.6%)
Previous MI, *n* (%)	13 (6.9%)	4 (6.6%)
Previous PCI, *n* (%)	17 (9.0%)	5 (8.2%)

*Clinical presentation*		
Stable angina, *n* (%)	8 (4.2%)	5 (8.2%)
Unstable angina, *n* (%)	18 (9.5%)	16 (26.2%)
ST-elevation myocardial infarction, *n* (%)	58 (30.7%)	18 (29.5%)
Non-ST-elevation myocardial infarction, *n* (%)	105 (55.6%)	22 (36.1%)

CAD = coronary artery disease; MI = myocardial infarction; PCI = percutaneous coronary intervention.

**Table 2:** Lesion and procedural characteristics for the entire population (*n* = 189) and for patients undergoing examination by quantitative coronary angiography (*n* = 61) at 9 months.

Variables	*n* = 189	*n* = 61
Number of lesions (*n*)	217	66

*Treated coronary artery*		
Left anterior descending artery, *n* (%)	105 (48.4%)	33 (50.0%)
Right coronary artery, *n* (%)	73 (33.6%)	22 (33.3%)
Left circumflex artery, *n* (%)	38 (17.5%)	11 (16.7%)
Left main, *n* (%)	1 (0.5%)	0 (0)

*Lesion classification (ACC/AHA score)*		
Type A, *n* (%)	6 (2.8%)	4 (6.1%)
Type B1, *n* (%)	30 (13.8%)	17 (25.8%)
Type B2, *n* (%)	108 (49.8%)	30 (45.5%)
Type C, *n* (%)	73 (33.6%)	15 (22.7%)
Total occlusion, *n* (%)	69 (31.8%)	14 (21.2%)
Total number of stents, (*n*)	230	76
Number of stents per patient (mean ± SD, mm)	1.22 ± 0.47	1.34 ± 0.60
Number of stents per lesion (mean ± SD, mm)	1.05 ± 0.29	1.12 ± 0.45
Average stent length (mean ± SD, mm)	24.88 ± 7.72	15.94 ± 7.95
Average stent diameter (mean ± SD, mm)	3.05 ± 0.34	3.15 ± 0.45
Predilation, *n* (%)	187 (98.9%)	59 (96.7%)
Postdilation, *n* (%)	127 (67.2%)	34 (55.7%)
Lesion success (%)	100%	100%
Device success (%)	100%	100%
Procedure success (%)	100%	100%

ACC/AHA = American College of Cardiology/American Heart Association.

**Table 3:** Results of quantitative coronary angiography analysis at preprocedure, postprocedure, and 9-month follow-up (*n* = 61 patients).

	In-segment	In-stent
*Preprocedure*		
Reference vessel diameter (mm)	2.42 ± 0.49	—
Diameter stenosis (%)	69.54 ± 18.81	—
Minimal lumen diameter (mm)	0.75 ± 0.53	—
Lesion length (mm)	9.70 ± 6.94	—

*Postprocedure*		
Reference vessel diameter (mm)	2.56 ± 0.44	2.65 ± 0.43
Diameter stenosis (%)	21.90 ± 8.49	14.07 ± 7.40
Minimal lumen diameter (mm)	2.01 ± 0.46	2.27 ± 0.39
Acute gain (mm)	1.26 ± 0.52	1.52 ± 0.53

*9-month follow-up*		
Reference vessel diameter (mm)	2.42 ± 0.43	2.49 ± 0.41
Diameter stenosis (%)	22.00 ± 8.65	16.14 ± 7.52
Minimal lumen diameter (mm)	1.90 ± 0.46	2.09 ± 0.44
Late lumen loss (mm)	0.11 ± 0.33	0.18 ± 0.23
Binary restenosis (%)	1.5	1.5

**Table 4:** Cumulative clinical outcomes up to 12 months (*n* = 189 patients).

Variables	30 days	6 months	12 months
Cardiac death, *n* (%)	1 (0.5%)	3 (1.6%)	3 (1.6%)
Noncardiac death, *n* (%)	0 (0%)	2 (1.1%)	6 (3.2%)
Myocardial infarction, *n* (%)	1 (0.5%)	1 (0.5%)	3 (1.6%)
Target lesion revascularization, *n* (%)	0 (0%)	2 (1.1%)	4 (2.1%)
Target vessel revascularization-non-TL, *n* (%)	0 (0%)	1 (0.5%)	3 (1.6%)
Stent thrombosis, *n* (%)	0 (0%)	0 (0%)	1 (0.5%)
Definite stent thrombosis, *n* (%)	0 (0%)	0 (0%)	1 (0.5%)
Target lesion failure, *n* (%)	2 (1.1%)	6 (3.2%)	10 (5.3%)

TL = target lesion.
